# Oncogenic Functions of Alternatively Spliced *MDM2-ALT2* Isoform in Retroperitoneal Liposarcoma

**DOI:** 10.3390/ijms252413516

**Published:** 2024-12-17

**Authors:** Fernanda Costas C. de Faria, Safiya Khurshid, Patricia Sarchet, Sayumi Tahara, Lucia Casadei, Valerie Grignol, Roma Karna, Sydney Rentsch, Nipin Sp, Joal D. Beane, Luciano Mazzoccoli, Matias Montes, Giovanni Nigita, Joe T. Sharick, Jennifer L. Leight, Federica Calore, Dawn S. Chandler, Raphael E. Pollock

**Affiliations:** 1 The James Comprehensive Cancer Center, Department of Surgery, Division of Surgical Oncology, The Ohio State University Wexner Medical Center, Columbus, OH 43210, USA; 2 Center for Childhood Cancer Research, Abigail Wexner Research Institute, Nationwide Children’s Hospital, Columbus, OH 43205, USA; 3 Division of Hematology, Department of Internal Medicine, The Ohio State University Comprehensive Cancer Research, Columbus, OH 43210, USA; 4 Department of Cancer Biology and Genetics, Comprehensive Cancer Center, The Ohio State University, Columbus, OH 43210, USA; 5 Department of Biomedical Engineering, College of Engineering, The Ohio State University, Columbus, OH 43210, USA

**Keywords:** MDM2 variants, MDM2-ALT2, MDM2-Full length, DDLPS, WDLPS, AKT, soft tissue sarcoma

## Abstract

Retroperitoneal liposarcoma (RPLPS) is one of the most common histologic subtypes of soft tissue sarcoma (STS). Complete surgical resection remains the mainstay treatment, while the high rate of locoregional recurrence constitutes the predominant cause of mortality. Well-differentiated (WDLPS) and dedifferentiated (DDLPS) liposarcoma are the most frequent subtypes of RPLPS and present amplified MDM2 gene as a hallmark. However, there are few reports evaluating the role of alternatively spliced MDM2 transcripts in RPLPS. In this study, we assessed MDM2-ALT2 expression levels in a cohort of RPLPS patients and evaluated the biological functions of the MDM2-ALT2 isoform in vitro in DDLPS cell lines. Using BaseScope™ and qPCR, we demonstrated that MDM2-Full Length (MDM2-FL) and MDM2-ALT2 expression levels were upregulated in RPLPS patient-derived tissue samples compared to normal adjacent to tumor tissue (NAT). DDLPS cells overexpressing MDM2-FL or MDM2-ALT2 had higher proliferation rates and increased migration and invasion capacities, as well as increased protein levels of p-AKT, mTOR, p70S6K, MMP2, and cJun. Simultaneous overexpression of MDM2-ALT2 and AKT silencing showed that AKT inhibition impaired p-p70S6K and MMP2 protein increased levels and led to significantly decreased proliferation and migration rates compared to cells overexpressing MDM2-ALT2 only. Taken together, our data suggest that MDM2-ALT2 may promote RPLPS progression.

## 1. Introduction

Retroperitoneal liposarcoma (RPLPS) is one of the most common histologic subtypes and locations of soft tissue sarcoma (STS) and is morphologically classified into well-differentiated (WDLPS), dedifferentiated (DDLPS), pleomorphic, and myxoid varieties [1,2]. WDLPS and DDLPS account for approximately 80% of RPLPS and can often simultaneously or sequentially coexist within the same patient [3,4]. RPLPS patients are usually diagnosed after tumors have reached a large size with involvement of major organs and visceral structures. Complete surgical excision remains the mainstay treatment strategy for RPLPS patients, but locoregional recurrence is still the predominant cause of RPLPS mortality, with overall survival of only 10% of patients at 10 years [3,5].

Both WDLPS and DDLPS are associated with chromosomal 12q13-15 region amplification, which includes *murine double minute 2* (*MDM2*), *cyclin-dependent kinase 4* (*CDK4*), and *HMGA2* [4,6,7] genes. Increased transcription and translation of the *MDM2* gene is a hallmark of WDLPS and DDLPS. The resultant increased MDM2 oncoprotein is functionally associated with inactivation of tumor suppressor gene *TP53*, which is rarely mutated in DDLPS [8]. Previously, we have reported that genomic *MDM2* amplification correlates with clinical outcomes in DDLPS cohorts [6]. However, there are few studies evaluating the role of alternatively spliced MDM2 transcripts in this disease.

Several MDM2 spliced variants have been documented in human tumors in addition to overexpression of MDM2 full length isoform (MDM2-FL), and alternatively spliced *MDM2* transcripts have been often associated with more aggressive disease and poor prognosis [9,10,11]. MDM2-ALT1 (MDM2-B), MDM2-ALT2 (MDM2-A), and MDM2-ALT3 (MDM2-C) are truncated polypeptide isoforms frequently overexpressed in many types of tumors, including RPLPS [12,13,14,15,16]. *MDM2* coding sequences are found in exons 3 through 12, encoding MDM2-FL. MDM2-ALT2 is an alternatively spliced isoform lacking exons 4-9, a region where the p53 binding site of MDM2 protein is located [12,17]. Most functions of MDM2-ALT2 evaluated to date are associated with its conserved C-terminal RING-finger binding domain, enabling it to bind to MDM2-FL and accounting for its E3 ligase properties [10,12,13]. So far, MDM2-ALT2 studies have presented seemingly contrasting findings, demonstrating that MDM2-ALT2 can inhibit tumor growth in a p53-dependent manner as well as enhancing transformation and displaying p53-independent oncogenic activity in a context-dependent manner [12,13,17].

In this study, we assessed for the first time *MDM2-ALT2* expression levels in a cohort of RPLPS patients. Moreover, we evaluated MDM2-ALT2’s biological functions in vitro in patient-derived DDLPS cell lines seeking to further elucidate the role of this alternatively spliced MDM2 isoform in RPLPS.

## 2. Results

### 2.1. MDM2 Splicing Variants Are Expressed in RPLPS Tumors

**Figure 1:**
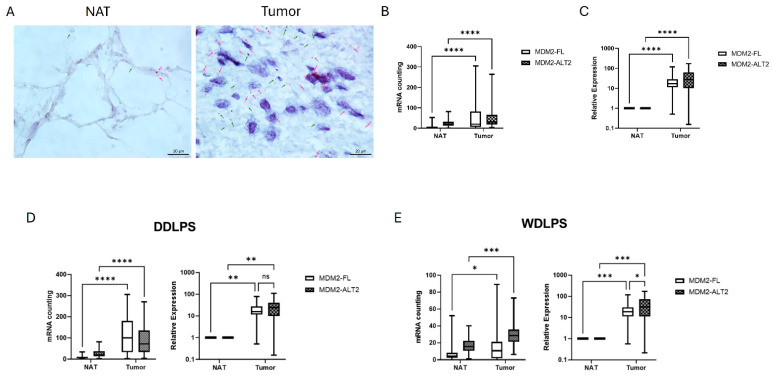
RPLPS patient tissue samples express *MDM2-ALT2*. (**A**) Representative BaseScope™ slides from WDLPS FFPE tissue samples showing *MDM2-FL* (pink dots/arrows) and *MDM2-ALT2* (green dots/arrows) mRNA in tumor tissue and matched normal adjacent tissue (NAT) (N = 7 patients). Magnified 63×. (**B**) Quantification of *MDM2-FL* and *MDM2-ALT2* mRNA from BaseScope™ in tumor and NAT tissues (N = 7 patients, **** *p* < 0.001). (**C**) Relative expression of *MDM2-FL* and *MDM2-ALT2* mRNA determined by qPCR using fresh frozen RPLPS tumor tissues in comparison to NAT tissues (N = 38 patients, **** *p* < 0.0001). (**D**) *MDM2-FL* and *MDM2-ALT2* mRNA quantification by BaseScope™ in DDLPS FFPE tumor and NAT tissues (N = 4 patients, **** *p* < 0.0001) (left panel) and relative expression assessed by qPCR of *MDM2-FL* and *MDM2-ALT2* in fresh frozen DDLPS tumor tissue in comparison to NAT (N = 14 patients, *MDM2-FL* ** *p* = 0.0036; *MDM2-ALT2* ** *p* = 0.0081) (right panel). (**E**) *MDM2-FL* and *MDM2-ALT2* mRNA quantification by BaseScope™ in WDLPS FFPE tumor and NAT tissues (N = 3 patients, * *p* = 0.013 and *** *p* < 0.001) (left panel); relative expression by qPCR of *MDM2-FL* and *MDM2-ALT2* in fresh frozen WDLPS tumor tissue in comparison to NAT (N = 24 patients, * *p* = 0.0164; *MDM2-FL* *** *p* = 0.0008; *MDM2-ALT2* *** *p* = 0.0004) (right panel), ns—not significant.

There are a small number of studies investigating the expression of different MDM2 isoforms in RPLPS patients. Previously, our group has demonstrated that IL6 signaling blockade modulated MDM2 isoforms, leading to a decrease in *MDM2-FL* and *MDM2-ALT2* levels (manuscript submitted). Therefore, we evaluated mRNA levels of both *MDM2-FL* and *MDM2-ALT2* isoforms in RPLPS tumors. Using two different methodologies, we were able to detect both isoforms in a cohort of RPLPS patient tissue samples. First, we used formalin-fixed paraffin-embedded (FFPE) tumor and normal adjacent to tumor (NAT) paired tissue specimens (n = 7) for an mRNA in situ hybridization methodology (BaseScope™). After probe hybridization, both *MDM2-FL* and *MDM2-ALT2* isoforms were significantly upregulated in tumor tissue in comparison to NAT (Figure 1A,B). We further confirmed this observation in a larger cohort of RPLPS patient tissue samples using qPCR (n = 38). Our relative expression analysis demonstrated that tumor tissues presented significant higher expression levels of *MDM2-FL* and *MDM2-ALT2* in comparison to NAT (Figure 1C). Based on these data, we continued our analysis by stratifying the RPLPS tissue samples by DDLPS and WDLPS histological subtypes. Both BaseScope™ and qPCR relative expression analysis demonstrated that *MDM2-FL* and *MDM2-ALT2* isoforms were significantly overexpressed in DDLPS and WDLPS tumor tissue in comparison to NAT (Figure 1D,E). Interestingly, WDLPS tissue samples demonstrated significantly higher levels of *MDM2-ALT2* in comparison to *MDM2-FL* (*p* = 0.0164). Genomic profiling of DDLPS compared to WDLPS demonstrated differences within these subgroups [18]. Therefore, we compared *MDM2-FL* and *MDM2-ALT2* mRNA expression levels in DDLPS versus WDLPS. However, qPCR did not indicate any statistically significant difference between the two subgroups (Supplementary Figure S1). Taken together, our data show that the *MDM2-ALT2* isoform may play a role in this challenging disease.

### 2.2. MDM2-ALT2 Contributes to Increased Oncogenic Phenotype in DDLPS Cell Lines

**Figure 2:**
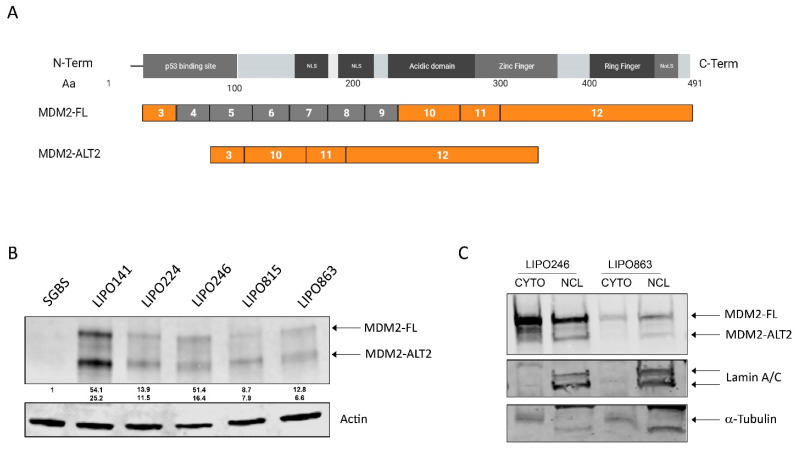
Alternative spliced MDM2 isoforms expression in RPLPS cell lines. (**A**) schematic illustration of *MDM2-FL* and *MDM2-ALT2* structures. The exons depicted in orange are present in MDM2-ALT2 and the exons in gray are the ones skipped in the FL isoform to generate MDM2-ALT2. (**B**) MDM2-FL (≈90 KDa) and MDM2-ALT2 (≈75 KDa) protein levels in a panel of DDLPS cell lines and in pre-adipocyte cell line, SGBS. (**C**) cellular localization of MDM2-FL and MDM2-ALT2 proteins in DDLPS cell lines. Images are representative of three independent experiments.

Based on the findings obtained from the RPLPS tissue samples, we extended our study to the protein expression and biological functions of the MDM2 isoforms, focusing on MDM2-ALT2 (Figure 2A). First, we evaluated the protein levels of MDM2-FL (≈90 KDa) and MDM2-ALT2 (≈75 KDa) in a panel of patient-derived DDLPS cell lines and the pre-adipocyte cell line SGBS, used as a control. We observed that all DDLPS cell lines expressed both MDM2-FL and MDM2-ALT2 at different levels; in pre-adipocyte cells SGBS, instead, both MDM2-FL and MDM2-ALT2 were barely detected (Figure 2B and Figure S2). We also observed that MDM2-ALT2 was more prevalent in the nucleus than in the cytoplasm of DDLPS cells (Figure 2C).

**Figure 3:**
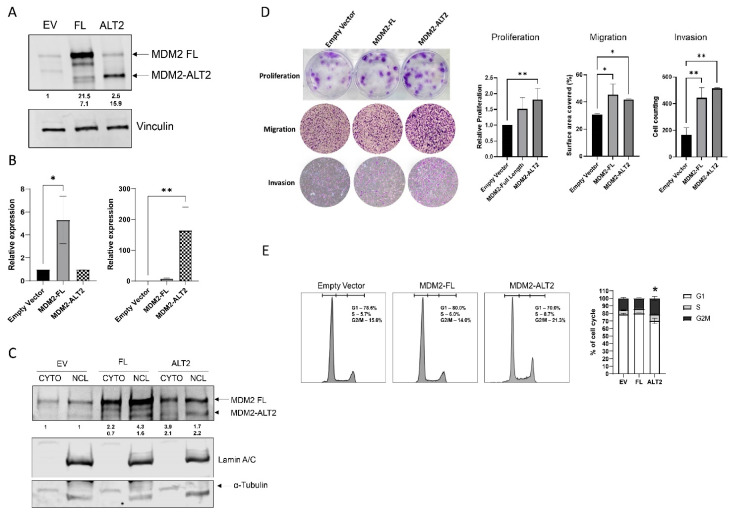
MDM2-ALT2 overexpression promotes an oncogenic phenotype in LIPO863 cells. (**A**) MDM2-FL and MDM2-ALT2 protein levels in LIPO863 detected by Western Blot 72 h after transfection. (**B**) Relative expression levels of *MDM2-FL* (* *p* = 0.04) and *MDM2-ALT2* (** *p* < 0.001) mRNA assessed by qPCR in transfected cells. (**C**) Cellular localization of MDM2-FL and MDM2-ALT2 proteins after transfection (72 h). (**D**) Representative images and relative quantification of proliferation (** *p* = 0.05), migration (* *p* = 0.04), and invasion (** *p* = 0.003) assays on LIPO863 cells after MDM2-FL or MDM2-ALT2 overexpression (72 h). (**E**) Cell cycle analysis of MDM2-FL or MDM2-ALT2-overexpressing cells in comparison to empty vector (EV) (* *p* = 0.05). Images are representative of three independent experiments.

We then overexpressed MDM2-FL or MDM2-ALT2 isoforms in DDLPS cell lines LIPO863 (Figure 3A,B) and LIPO246 and SGBS (Supplementary Figure S3A,B). Following overexpression, MDM2-ALT2 continued to localize in the nucleus of DDLPS cells (Figure 3C), and cells demonstrated higher proliferation rates and increased migration and invasion abilities in both DDLPS cell lines (Figure 3D and Figure S3A), but not for SGBS (Supplementary Figure S3B). Cell cycle distribution of LIPO863 overexpressing MDM2-ALT2 demonstrated increased percentages of cells in G2/M phase in comparison to the empty vector control (EV) (Figure 3E).

**Figure 4:**
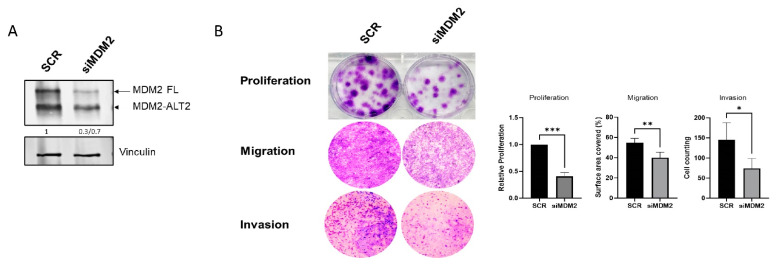
MDM2 isoform inhibition reverses oncogenic phenotype in LIPO863 cells. (**A**) MDM2-FL and MDM2-ALT2 protein levels after siRNA transfection (48 h) detected by Western Blot. (**B**) Representative images and relative quantification of proliferation (*** *p* = 0.0005), migration (** *p* = 0.0096), and invasion (* *p* = 0.0296) assays of cells after MDM2 downregulation (48 h). Images are representative of three independent experiments.

We further evaluated the biological effects of MDM2 isoform inhibition in RPLPS cells. For this purpose, we downregulated MDM2 isoforms using siRNA transient transfection (Figure 4A): the results showed a significantly decrease in proliferation, migration, and invasion (Figure 4B), validating our previous findings.

### 2.3. MDM2-ALT2 Interactions Are Not Dependent on p53 or MDM2-FL

In this context, we next investigated whether molecular pathways already described in RPLPS or associated with functions reported to be modulated by MDM2 isoforms overexpression were involved in the observed biological effects. First, we evaluated if p53 was being modulated after MDM2-FL or MDM2-ALT2 isoforms’ overexpression. We did not observe any significant differences in p53 protein levels (Supplementary Figure S4A) and there were no statistically significant changes in mRNA expression of p53 targets *PCNA*, *Bax*, *WIP1*, *PUMA*, *P21*, and *Gadd45a* in any of the cell lines tested (Supplementary Figure S4B). Our data suggest that the observed effects are p53-independent. There were also no differences in MAPK/ERK levels or phosphorylation in cells overexpressing MDM2-ALT2 in comparison to control (Supplementary Figure S4C). Some studies have demonstrated that MDM2 isoforms can heterodimerize and, therefore, enhance or inhibit MDM2-FL activities [19]. Thus, we sought to evaluate whether MDM2-ALT2 and MDM2-FL directly interacted in transfected DDLPS cells overexpressing each specific isoform. However, after immunoprecipitating MDM2-FL, we did not detect the presence of MDM2-ALT2 in the protein pull down (Supplementary Figure S4D). Taken together, our data suggest that MDM2-ALT2 does not interact directly with MDM2-FL and, therefore, its downstream function may be independent of their interaction.

Several classes of MDM2 inhibitors are currently under investigation, with most presenting the p53 binding site of MDM2 protein as the main target of inhibition [5]. A possible explanation for the lower rates of therapeutic success may be associated with the presence of different MDM2 splicing variants lacking p53 binding site. Therefore, we evaluated the role of MDM2 isoforms in response to MDM2 inhibitor treatment. MDM2 overexpressing-LIPO863 were treated with increasing doses of the MDM2 inhibitor SAR405838 for 24 h, 48 h, and 72 h, followed by cell viability assessment. There were no significant differences between MDM2-ALT2 overexpressing cells and control; however, after 24 h of incubation with SAR405838, we observed that MDM2-FL-overexpressing cells were more sensitive to SAR405838 than other conditions (Supplementary Figure S5A). We have also evaluated MDM2 isoforms and p53 protein levels after treatment with MDM2 inhibitor. Interestingly, we observed increased expression of MDM2-FL and MDM2-ALT2 in a dose-dependent manner with no significant changes in p53 levels after 24 h, 48 h, and 72 h treatment of LIPO863 with SAR405838 (Supplementary Figure S5B), suggesting that, in the presence of SAR405838, the cells increase MDM2 isoforms expression perhaps in an attempt to overcome the effects of the inhibitor.

### 2.4. MDM2 Isoforms Overexpression in DDLPS Cells Leads to the Activation of PI3K/AKT/mTOR Pathway

**Figure 5:**
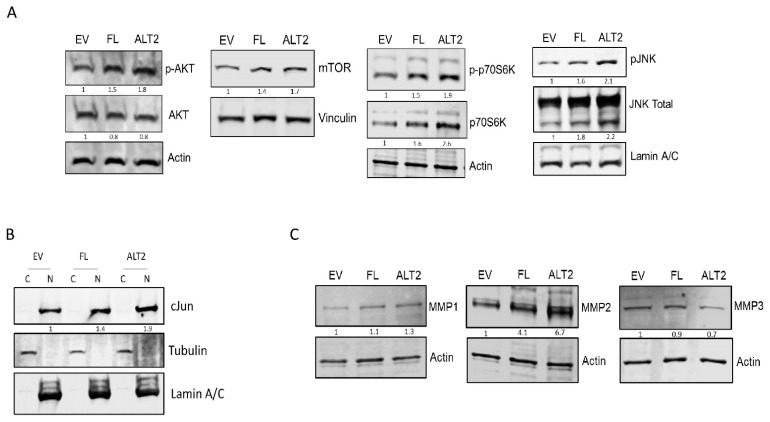
Molecular pathways activated by MDM2-ALT2 in LIPO863 cell line. (**A**) Western Blot analysis of AKT, p-AKT, mTOR, p70S6K, p-p70S6K, JNK, and cJun protein levels in LIPO863 overexpressing MDM2-FL or MDM2-ALT2 isoforms. (**B**) Cellular localization of cJun protein after MDM-FL or MDM2-ALT2 overexpression. (**C**) MMP1, MMP2, and MMP3 protein levels observed 72 h after transfection.

Several studies have reported PI3K/AKT/mTOR signaling pathway as being activated in RPLPS [2,20,21]. Therefore, we investigated the possible modulation of this pathway by MDM2 isoforms. Our data demonstrated an increase in AKT phosphorylation (Ser473) in DDLPS cell lines after MDM2-FL or MDM2-ALT2 overexpression, more prominent in MDM2-ALT2 overexpressing cells (Figure 5A and Figure S6A). Consistently, mTOR protein levels were also increased in cells overexpressing MDM2-ALT2. Furthermore, increased protein levels and increased phosphorylation of p70S6K were also observed in transfected cells, but at higher levels in the cells transfected with the MDM2-ALT2 isoform (Figure 5A). The JNK MAPK family was also found to be upregulated by MDM2 isoforms, especially by MDM2-ALT2 (Figure 5A). DDLPS cells overexpressing MDM2-FL or MDM2-ALT2 isoforms presented higher protein levels and increased phosphorylation of cJun, as well as an increase in nuclear cJun (Figure 5A,B). cJun has been reported to be amplified in RPLPS patients [5,22,23] and shares downstream targets with PI3K/AKT/mTOR axis, corroborating our findings.

Metalloproteinases (MMPs) are a family of endopeptidases that mediate several physiological and pathological cellular processes such as proliferation, migration, and invasion by degrading a variety of extracellular matrix (ECM) proteins or by regulating growth factors, receptors, and immune responses [24]. MMPs’ transcription can be activated by NF-κB, cJun, and AKT pathways [25]. Consequently, we further verified whether MMPs’ expression could be modulated by different MDM2 isoforms. Western Blot analysis revealed increased levels of MMP1 and MMP2, but not MMP3, in DDLPS cells overexpressing MDM2-FL or especially MDM2-ALT2 (Figure 5C). However, upregulation of MMP2 was not associated with increased activity of the metalloproteinase in conditioned media, as assessed by gelatin zymography (Supplementary Figure S6B), suggesting the possibility that MMP2 might be exerting intracellular functions in the presence of MDM2 isoforms, rather than acting on the degradation of proteins in the extracellular matrix.

**Figure 6:**
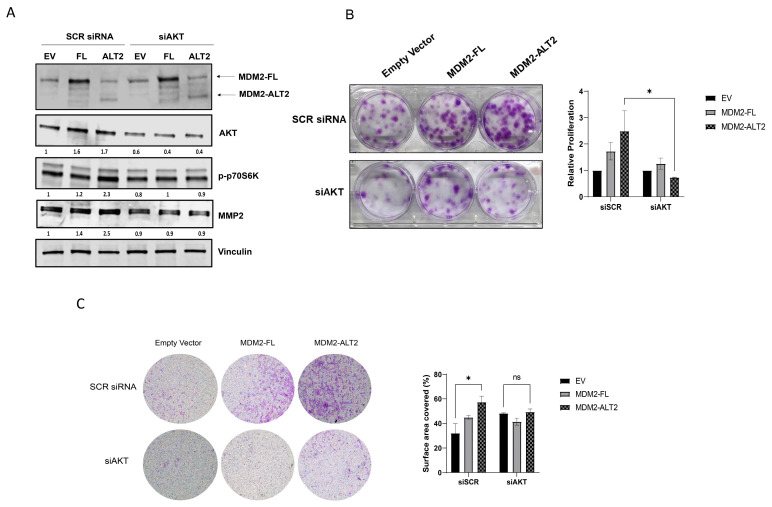
The oncogenic phenotype of DDLPS cells overexpressing MDM2-ALT2 is reversed after AKT inhibition. (**A**) Western Blot analysis of AKT, p-p70S6K, and MMP2 in LIPO863 co-transfected with MDM2-FL or MDM2-ALT2 and siAKT for 72 h. (**B**) Representative image of clonogenic assay and relative proliferation quantification of LIPO863 after co-transfection (* *p* = 0.007). (**C**) Representative image of migration assay and percentage of surface area covered graph of LIPO863 after co-transfection (* *p* = 0.015). Images are representative of three independent experiments, ns—not significant.

Having demonstrated that DDLPS cells overexpressing MDM2-ALT2 present modulated PI3K/AKT/mTOR pathway, we impaired AKT expression in cells transfected with MDM2 isoforms. We co-transfected DDLPS cell line LIPO863 with MDM2-FL or MDM2-ALT2 and a small interference RNA for AKT (siAKT) (Figure 6A). After co-transfection, we observed that AKT inhibition prevented p-p70S6K and MMP2 protein levels from increasing, as well as decreased proliferation and migration rates of cells overexpressing MDM2-ALT2 (Figure 6A–C). Taken together, our data suggest a possible AKT-dependent mechanism in which MDM2-ALT2 promotes proliferation and migration in DDLPS cells.

## 3. Discussion

*MDM2* is considered a main driver gene of WDLPS and DDLPS since it promotes tumorigenesis by inhibiting p53 transcriptional activity. Due to its E3 ubiquitin ligase activity, MDM2 mediates p53 ubiquitination and subsequently proteasome degradation, thereby preventing p53 target activation [5,26]. *TP53* is rarely mutated in DDLPS patients and cell lines [6,8]. Previously, our group demonstrated that both genomic amplification and mRNA expression of MDM2 were associated with reduced overall survival and shortened time to recurrence [6]. Also, DDLPS-derived extracellular vesicle *MDM2* cargo led to downregulation of p53 activity and release of active MMP2 in recipient pre-adipocyte cells commonly found in RPLPS tumor microenvironment, thereby possibly contributing to the establishment of a premetastatic niche [27]. Research on the mechanisms involving *MDM2* amplification in RPLPS has mainly focused on genomic amplification or mRNA expression of MDM2-FL. MDM2-ALT1 and MDM2-ALT2 were first described to be expressed in soft tissue sarcoma samples, including liposarcomas, in 2001 by Bartel and colleagues, but without reporting correlation with MDM2 amplification [28]. Additionally, MDM2-ALT3 was detected in liposarcoma tumor samples by Okoro and colleagues in 2013 [14], and while the expression of MDM2-ALT2 correlated with the MDM2 overexpression in SJSA and MANCA cell lines, this correlation was not tested in liposarcoma or breast cancer patient samples used in the study [14]. Understanding the overexpression of MDM2 in cancers, particularly sarcomas, as well as their p53 status is critical for therapeutic implications. However, there are few studies examining the role and expression of alternatively spliced isoforms of MDM2 in RPLPS [14,15,29,30]. These factors highlight the need for further studies to investigate the role of MDM2 splice variants in various contexts. Given our findings on the significance of MDM2-ALT2, examining its correlation with MDM2 amplification in RPLPS could offer promising avenues for potential clinical applications.

MDM2 isoforms contribute to cancer proteome diversity and support oncogenic transformation [10]. MDM2-ALT1, MDM2-ALT2, and MDM2-ALT3 are constitutively expressed in different types of tumors and have been correlated with tumorigenesis and high-grade metastatic disease [14,31,32,33,34]. Indeed, a variety of *MDM2* alternatively spliced mRNAs have been described, with many MDM2 isoforms lacking the p53-binding domain, suggesting that their oncogenic activity extends beyond p53 degradation. There are numerous MDM2-interacting proteins and several proposed p53-independent mechanisms by which MDM2 can affect tumorigenesis. Examples include ubiquitination with consequent proteasomal degradation of various transcription factors and tumor suppressor proteins, as well as induction of genomic instability and DNA damage [7,11,35]. *MDM2* amplification detected by fluorescence in situ hybridization (FISH) is commonly used as a gold standard RPLPS diagnostic method due to its high sensitivity and specificity. However, FISH experimental implementation requires experienced personnel and is time-consuming [36]. Immunohistochemistry for MDM2 protein expression is normally used to confirm FISH data, but it has lower specificity and sensitivity [37]. New platforms able to detect mRNA in FFPE tissue samples, such as BaseScope™, enable studies of alternatively spliced mRNA isoforms expression in a variety of tissues. Here, we demonstrate for the first time increased levels of *MDM2-FL* and *MDM2-ALT2* mRNA in WDLPS and DDLPS patient tumor samples in comparison to NAT tissue. We are now expanding our RPLPS patient-derived tissue cohort to evaluate the impact of MDM2 splicing variants in the recurrence of RPLPS patients. Further studies exploring the role of MDM2-ALT2 and its correlation with MDM2 amplification in RPLPS can lead the way to potential clinical use of this isoform.

Earlier studies have demonstrated that alternatively spliced MDM2 variants can be as oncogenic as MDM2-FL in vivo and have p53-independent functions in tumorigenesis, as observed in p53-deficient mice [11,38]. However, studies evaluating MDM2 splicing variant functions regarding tumor development have been inconclusive. MDM2-ALT2 has been described as associated with both pro-oncogenic and growth-inhibitory phenotypes and perinatal lethality; it may also be important in the development of highly undifferentiated, aggressive mammary tumors [12,17]. Here, we show that DDLPS cells overexpressing MDM2-ALT2 are characterized by increased proliferation, migration, and invasion rates. Strikingly, these effects were reversed once the isoforms were downregulated. These results point towards MDM2-ALT2 tissue-specific functions and highlight a novel role for MDM2-ALT2 in RPLPS disease progression.

The oncogenic activity of alternatively spliced MDM2 isoforms that cannot bind directly to p53 most likely require the cooperation of other proteins and pathways [35]. It is well known that MDM2 splicing variants can directly interact with MDM2-FL, perhaps thereby helping regulate the p53 pathway [12,29,35,39]. However, other entities interacting with MDM2 isoforms, especially MDM2-ALT2, are yet to be clarified. Here, we suggest that MDM2-ALT2 expression in DDLPS cell lines does not interfere with either p53 expression or its targets. Also, MDM2-ALT2 did not directly interact with MDM2-FL, suggesting that overexpressed MDM2-ALT2-induced effects may be associated with p53-independent MDM2 functions. We are now expanding our studies to better elucidate the role of MDM2-ALT2 in p53 pathway and their interactions.

A better understanding of the role of MDM2 alternative splicing in RPLPS and in response to MDM2 inhibitors could also provide untapped benefits for the development of future MDM2-targeting compounds. MDM2 inhibitors are small molecules exhibiting potent abilities to disrupt the MDM2-p53 axis. The mechanism of action of these compounds relies on binding to N-terminus region of MDM2, destabilizing p53-MDM2 protein–protein interactions, thereby leading to p53 stabilization and activation of downstream pathways. However, the best clinical response achieved by using those inhibitors is stable disease. Additionally systemic activation of p53 by MDM2 inhibitors has led to severe side effects, specially associated to bone marrow suppression [40,41,42]. To address these issues, new molecules have been developed with improved pharmacokinetics properties, such as Alrizomadlin, designed for improved stability, and Brigimadlin, an MDM2 inhibitor that shows favorable systemic clearance profile, solubility, and permeability [40,43,44,45]. Nevertheless, the mechanism of action of the new generation of MDM2 inhibitors remains unchanged. Therefore, MDM2 isoforms lacking a p53 docking site are unlikely to be appropriately targeted by this class of compounds. In fact, Bozzi and colleagues demonstrate that the MDM2 inhibitor Nutlin-3A has a binding pocket particularly well suited to binding the MDM2-FL protein. Their in silico analysis further revealed that Nutlin-3A was only predicted to bind to MDM2-B and MDM2-C very weakly compared to MDM2-FL. The in vitro and in silico findings of this study suggest that MDM2 isoforms will influence Nutlin-3A sensitivity and resistance in WDLPS and DDLPS [30], further supporting the clinical role that MDM2 isoforms potentially play in this context.

It has been reported that Jun amplifications are associated with DDLPS and WDLPS containing a dedifferentiated component; these are usually associated with a worse prognosis compared to WDLPS [21,23]. Here, we suggest that MDM2-ALT2 expression induces JNK/cJun activation by increasing JNK and cJun phosphorylation levels and also promotes cJun nuclear localization. This regulatory pathway may be contributing to the more aggressive phenotype observed in the MDM2-ALT2 overexpressing cells and needs to be further evaluated.

The role of the AKT/mTOR/p70S6K axis is not completely understood in RPLPS. Activated forms of AKT were identified in liposarcoma cell lines and WDLPS and DDLPS patient samples. Also, AKT2 is constitutively active in mesenchymal stem cells and induces WDLPS formation. Furthermore, cell lines derived from WDLPS and DDLPS patients have impaired cellular viability after AKT pathway inhibition [20,21,46]. Here, we show increased levels of AKT phosphorylation after MDM2-ALT2 overexpression along with mTOR and its downstream target protein p70S6K, suggesting that this pathway might play an important role in the effects exerted by MDM2-ALT2 overexpression. Extending our knowledge on RPLPS tumorigenesis and drivers beyond MDM2-p53 axis offers opportunities to design different therapeutic strategies by combining two or more targeted drugs. Some studies have demonstrated that the use of everolimus, a selective mTOR inhibitor, in combination with ribociclib (a kinase inhibitor) or bortezomib (a proteosome inhibitor), can enhance inhibition of tumor growth and lead to stable disease, increasing overall survival [47,48]. In our study, we demonstrated that the mTOR pathway is modulated by MDM2-ALT2 upregulation, suggesting this pathway as a possible therapeutic target when high MDM2-ALT2 levels are detected.

Previously, our group demonstrated that DDLPS-derived extracellular vesicles containing *MDM2* as cargo induced MDM2 expression and MMP2 activation in pre-adipocytes [27]. MMP2 is a gelatinase that degrades ECM proteins and is commonly associated with pathological cellular processes such as proliferation, migration, and invasion [24]. An important mechanism by which PI3K/AKT/mTOR/p70S6K plays a role in cell migration and invasion is by triggering the expression of MMP2 and MMP9 [49,50,51]. In this context, we evaluated MMP2 expression and activity in DDLPS cells overexpressing MDM2-ALT2. MMP2 levels were increased by MDM2-ALT2 overexpression, but minimal extracellular MMP2 activity was detected. However, MMP’s biological function is not limited to the regulation of ECM. In osteosarcoma cells, nuclear MMP2 has been associated with cancer proliferation induction via histone H3 cleavage, and inactivation of the intracellular MMP2/p38 pathway suppressed angiogenesis and tumor growth in ovarian cancer cells [52,53,54]. We examined the role of the AKT/mTOR/p70S6K/MMP2 axis in MDM2-ALT2-overexpressing cells by inhibiting AKT expression. Our data showed that AKT inhibition led to decreased phosphorylation of p70S6K and MMP2 protein in cells overexpressing MDM2-ALT2. Also, cellular proliferation and migration were impaired in cells overexpressing MDM2-ALT2 after AKT inhibition by siRNA. We are now extending these studies to better understand the crosstalk between AKT and MDM2-ALT2 to consider whether these effects could be due to MMP2 induction.

## 4. Materials and Methods

### 4.1. Patient Samples

The retrospective cohort study included 38 RPLPS tumors and normal adjacent to tumor (NAT) fresh-frozen and formalin-fixed paraffin-embedded (FFPE) tissues from a tissue bank at The Ohio State University Wexner Medical Center. This study was conducted with the approval of The Ohio State University Wexner Medical Center Institutional Review Board with written informed consent of the participants.

### 4.2. BaseScope™ RNA In-Situ Hybridization Assay

To detect MDM2 isoforms in FFPE samples, we employed the BaseScope™ RNA ISH assay (Advanced Cell Diagnostics, Newark, CA, USA) according to manufacturer’s instructions and using BaseScope™ custom-designed probes BA-Hs-MDM2-1zz-st (targeting 664-710 of NM_002392.6) and BA-Hs-MDM2-A-O1-Junc (targeting 50-92 of U33199.1). Slides were counterstained with 50% Hematoxylin staining solution. Target probe signal was evaluated under a standard bright field Zeiss Axioskop microscope, and quantification was performed by Colour Deconvolution using FIJI-ImageJ v1.53f51, as described previously [55,56].

### 4.3. Real-Time PCR (qPCR)

RNA was extracted using RNeasy^®^ Kit (Qiagen, Germantown, MD, USA), following manufacturer’s instructions, and quantified using NanoDrop™ (ThermoFisher Scientific, Waltham, MA, USA). Samples of 180 ng of RNA were used for the reverse transcription (RT) reactions carried out using High-Capacity cDNA Reverse Transcription Kit (Applied Biosystems, Thermo Scientific, Waltham, MA, USA). Quantitative Real-time PCR (qPCR) reactions were carried out using Taqman Fast Advanced Master Mix (Applied Biosystems, Thermo Scientific). MDM2-FL (Hs01066941_m1, Thermo Scientific) and MDM2-ALT2 probe, a custom assay that covered the junction point between exons 3 and 10 of *MDM2-ALT2* sequence, were used. Lrp10 (Hs01047362_m1, Thermo Scientific) and Pgk1 (Hs99999906_m1, Thermo Scientific) were used to normalize qPCR.

### 4.4. Cell Culture and Regents

Human DDLPS cell lines LIPO224, LIPO246, LIPO815, and LIPO863 were generated at our laboratory as previously described [57], and LIPO141 was obtained from Dr. Jonathan Fletcher (Brigham & Women’s Hospital, Boston, MA, USA). DDLPS cell lines were cultured in DMEM (Gibco^®^; Thermo Fisher Scientific, Inc.) supplemented with 10% heat inactivated fetal bovine serum (FBS) (Gibco; Thermo Fisher Scientific, Inc.). Pre-adipocyte (Pre-a) cell line SGBS [58] was maintained in DMEM-Nutrient Mixture F-12 (DMEM-F12) (Gibco^®^; Thermo Fisher Scientific, Inc.) supplemented with 33 μmol/L biotin, 17 μmol/L pantothenate, and 10% non-heat inactivated FBS. All cell lines were cultured in a humidified atmosphere at 37 °C with 5% CO_2_, authenticated by STR, and periodically tested negative for mycoplasma. Cells were allowed to adhere as a monolayer to culture plates overnight before experimental analysis.

### 4.5. Overexpression and Silencing of MDM2 Isoforms and siRNA-AKT Co-Transfection

Amounts of 3 × 10^5^ cells for LIPO863 and 4 × 10^5^ cells for LIPO246 and SGBS were seeded per well in 6-well plates for MDM2 isoforms overexpression experiments. On the following day, 2 μg of pCALL2-MDM2 Full Length (MDM2-FL) or pCALL2-MDM2-ALT2 (MDM2-ALT2) plasmids were transfected in a single well using 8 μL of Lipofectamine™ 3000 Transfection Kit (Invitrogen, Waltham, MA, USA) and incubated at 37 °C for 72 h. Small interference RNA for MDM2 (Invitrogen) transient transfection was performed by seeding LIPO863 (3 × 10^5^ cells per well) in 6-well plates. On the next day, 100 nM of MDM2 siRNA and negative control RNA (Invitrogen) were transfected to cells using 8 μL Lipofectamine™ RNAiMAX per well and incubated at 37 °C for 48 h. For co-transfection with small interference RNA for AKT (siAKT, Cell Signaling, Danvers, MA, USA), 3 × 10^5^ cells of LIPO863 were seeded in 6-well plates and co-transfected with 2 μg of pCALL2-MDM2-FL or pCALL2-MDM2-ALT2 plasmids and 100 nM of siAKT, simultaneously, using 8 μL of Lipofectamine™ 3000 Transfection Kit (Invitrogen) per reaction. Co-transfected cells were incubated at 37 °C for 72 h. After 48 h or 72 h, cells were washed with DPBS 1X (Gibco), harvested and centrifuged at 750× *g* for 3 min at room temperature, and counted to be seeded for subsequent experiments.

### 4.6. Proliferation Assay

A colony formation assay was used to assess proliferation by seeding 300 transfected cells in each well of a 6-well plate and cultured at 37 °C for 10–14 days until colonies were observed. After that, colonies were fixed with ethanol for 10 min and stained with 0.5% crystal violet solution (Thermo Fisher Scientific) for 1 h with gentle agitation at room temperature. In order to quantify the colonies, a spectrophotometric analysis of crystal violet-stained cells was performed by dissolving the colonies in 33% acetic acid and measuring the absorbance at 595 nm using microplate reader Cytation 3 (BioTek^®^, Winooski, VT, USA). The absorbance values were used to calculate the relative proliferation rate of the cells after transfections.

### 4.7. Migration and Invasion Assays

Migration and invasion assessment were performed using 8 μm trans-well ThinCerts™ migration chambers (Greiner Bio-One, Kremsmünster, Austria) and 8 μm Corning Matrigel Invasion Chambers (Corning, Corning, NY, USA), respectively. Amounts of 8 × 10^4^ and 10^5^ cells were seeded into the upper migration and invasion chamber, respectively, in 200 μL plain DMEM. DMEM with 5% FBS was the chemoattractant used for these experiments. Migration and invasion assay endpoints were set for 24 h and 48 h after seeding, respectively. At endpoint, cells were fixed with ethanol for 10 min and stained with 0.5% crystal violet solution for 1 h. For quantitative analysis, each invasion chamber was divided into 4 quadrants and the invaded cells were counted. The stained migration chambers were photographed, and the percentage of covered surface area of the chamber was calculated using ImageJ v1.53f51 software. Briefly, each image was converted into a binary image and cropped using the same dimensions parameters (*X* axis; *Y* axis; height; width), followed by a particle counting. All photographs were obtained using a 4× objective of an inverted brightfield EVOS™ XL Core microscope (Thermo Fisher Scientific, Waltham, MA, USA).

### 4.8. Sub-Cellular Protein Fractioning, Co-Immunoprecipitation and Western Blot

Cytoplasmatic and nuclear protein fractions were obtained using NE-PER™ Nuclear and Cytoplasmic Extraction Reagents (Thermo Fisher Scientific) following manufacturer’s instructions. Co-immunoprecipitation of MDM2-FL and MDM2-ALT2 proteins was performed coupling 7 μg of MDM2 antibody (SMP14 clone, Santa Cruz, Dallas, TX, USA) to 1.5 mg of magnetic beads. For that, Dynabeads^®^ Co-Immunoprecipitation Kit (Life Technologies, Carlsbad, CA, USA) was used, following manufacturer’s instructions. For Western Blot analysis, 25–30 μg of proteins were loaded into 4–20% Mini-PROTEAN^®^ Precast Gels (Bio-Rad, Hercules, CA, USA) and transferred using a Trans-Blot Turbo Transfer System (Bio-Rad). Membranes were incubated overnight at 4 °C with the following primary antibodies: MDM2 (D1V2Z, Cell Signaling); p53 (Santa Cruz); p-AKT (Ser473, Cell Signaling); AKT (Cell Signaling); p-ERK1/2 (Thr202/Tyr204, Cell Signaling); ERK (Cell Signaling); mTOR (Cell Signaling); p-p70S6K (Thr389, Cell Signaling); p70S6K (Cell Signaling); p-JNK (Thr183/Tyr185, Cell Signaling); JNK (Cell Signaling); p-cJun (Ser63, Cell Signaling); cJun (Cell Signaling); MMP1 (Cell Signaling); MMP2 (Cell Signaling); MMP3 (Cell Signaling); Lamin A/C (Santa Cruz); Actin (Santa Cruz); Tubulin (Santa Cruz); Vinculin (Santa Cruz). IRDye^®^ 800CW and IRDye^®^ 680RD (LI-COR, Inc., Lincoln, NE, USA) were used as secondary antibodies and fluorescence was detected using Odyssey CLx Imager (LI-COR, Inc.) and Image Studio v5.2 Software (LI-COR, Inc.).

### 4.9. Gelatin Zymography

Gelatin zymography was performed as described previously [27]. Briefly, 500 μL of conditioned medium derived from transfected LIPO863 cells was concentrated using Millipore’s (Burlington, MA, USA) Amicon^®^ Ultra-0.5 centrifugal filter units into 20 μL volume. Then, 5 μL of concentrated medium diluted in 25 μL of loading buffer was further loaded onto gelatin zymography gels (10% polyacrylamide, 0.1% gelatin) and electrophoresed for 2 h at 120 V at 4 °C. Gels were then washed in renaturing buffer and transferred to a developing buffer solution to be incubated overnight at 37 °C under gentle agitation. Gels were finally stained with 0.5% Coomassie Brilliant Blue (Thermo Fisher Scientific) for 2 h at room temperature. Images were captured using Odyssey CLx Imaging System (LI-COR, Inc.) and Image Studio Software (LI-COR, Inc.).

### 4.10. Cell Cycle Analysis

LIPO863 cells were transfected for 72 h, counted, and then seeded for cell cycle evaluation. After 72 h, cells were harvested, fixed, and stained with propidium iodide/Triton X-100 solution (0.3% Triton X-100, Bio-Rad; propidium iodide 50 μg/mL, Sigma-Aldrich Co., MO, USA) and RNAse (100 μg/mL, Sigma-Aldrich Co.). Cells were analyzed in Cytek Aurora flow cytometer (Cytek^®^ Biosciences, Fremont, CA, USA), and data were analyzed with FlowJo™ v10 software (BD Biosciences, Franklin Lakes, NJ, USA).

### 4.11. Cell Viability

Transfected LIPO863 cells were seeded at a density of 5 × 10^3^ cells per well of a 96-well plate. On the following day, serial dilutions of MDM2 inhibitor SAR405838 (Sanofi-Aventis, Bridgewater, NJ, USA) were prepared for cellular viability assessment, with a final concentration of DMSO not exceeding 0.1%. Cells were treated with SAR405838 for 24 h, 48 h, and 72 h, and cellular viability was assessed using CellTiter96^®^ Aqueous One Solution Cell Proliferation Assay (Promega, Madison, WI, USA) following manufacturer’s instructions. Absorbances were measured at 490 nm using microplate reader Cytation 3 (BioTek^®^, Winooski, VT, USA).

### 4.12. Statistical Analysis

Statistical analyses were performed using GraphPad Prism version 9.2.0 software and all values were stated as mean ± SD. For the BaseScope™ and Real-time PCR data analysis, two-way ANOVA was performed, and Spearman’s Rank Correlation Coefficient test was performed to compare BaseScope™ and qPCR. For biological assays data analysis, one-way ANOVA and a two-tailed Student’s *t*-test were utilized to evaluate statistical significance (*p* > 0.05).

## 5. Conclusions

In this study, we demonstrated for the first time increased levels of *MDM2-ALT2* mRNA in WDLPS and DDLPS patient tumor samples in comparison to NAT tissue. Overexpressing MDM2-ALT2 in DDLPS cells led to increased proliferation, migration and invasion rates compared to control condition, suggesting that MDM2-ALT2 may play a role in RPLPS progression. We further demonstrated that the AKT/mTOR/p70S6K/MMP2 axis might play an important role in the effects exerted by MDM2-ALT2 overexpression since AKT inhibition led to decreased phosphorylation of p70S6K and MMP2 protein as well as impaired cellular proliferation and migration in cells overexpressing MDM2-ALT2. Taken together, our data suggest that MDM2-ALT2 may play an oncogenic role in RPLPS, highlighting the importance of expanding the studies of MDM2 alternative splicing variants expression and functions in this highly lethal sarcoma.

## Data Availability

The data generated in this study are not publicly available so as to not compromise patient privacy or consent but are available upon reasonable request from the corresponding author.

## Supplementary Materials

The following supporting information can be downloaded at: https://www.mdpi.com/article/10.3390/ijms252413516/s1.
