# Supplementary material for: Oncogenic Functions of Alternatively Spliced MDM2-ALT2 Isoform in Retroperitoneal Liposarcoma
Source: Int J Mol Sci. 2024 Dec 17;25(24):13516. doi: 10.3390/ijms252413516 (PMC11676768; doi:10.3390/ijms252413516)
Supplement: Supplementary file 1 [file ijms-25-13516-s001.zip › ijms-3355345-supplementary.pdf]

Supplementary Materials

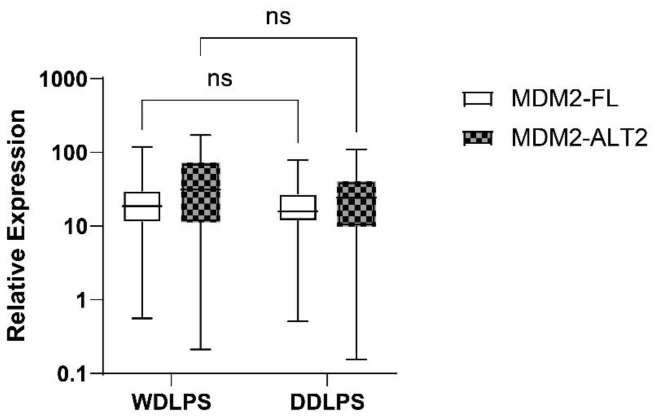

Supplementary Figure S1. Relative expression levels of MDM2 isoforms in different histological subtypes of RPLPS**.** *MDM2-FL* and *MDM2-ALT2* relative expressions were determined by qPCR in fresh frozen WDLPS tumor tissue in comparison to DDLPS samples (N=38 patients).

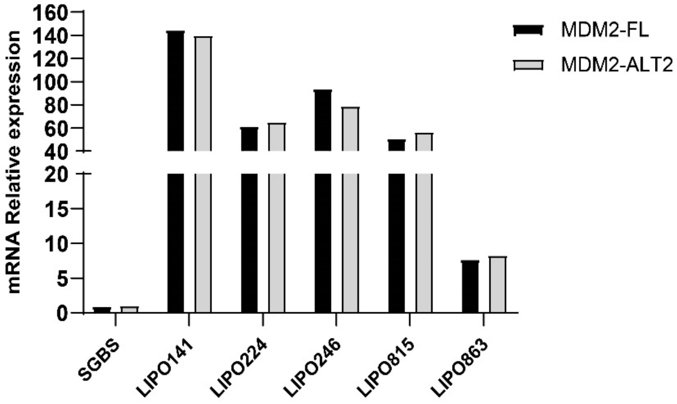

Supplementary Figure S2. Relative mRNA expression levels of MDM2-FL and MDM2-ALT2 determined by qPCR in a panel of DDLPS cell lines in comparison to pre-adipocyte cell line, SGBS.

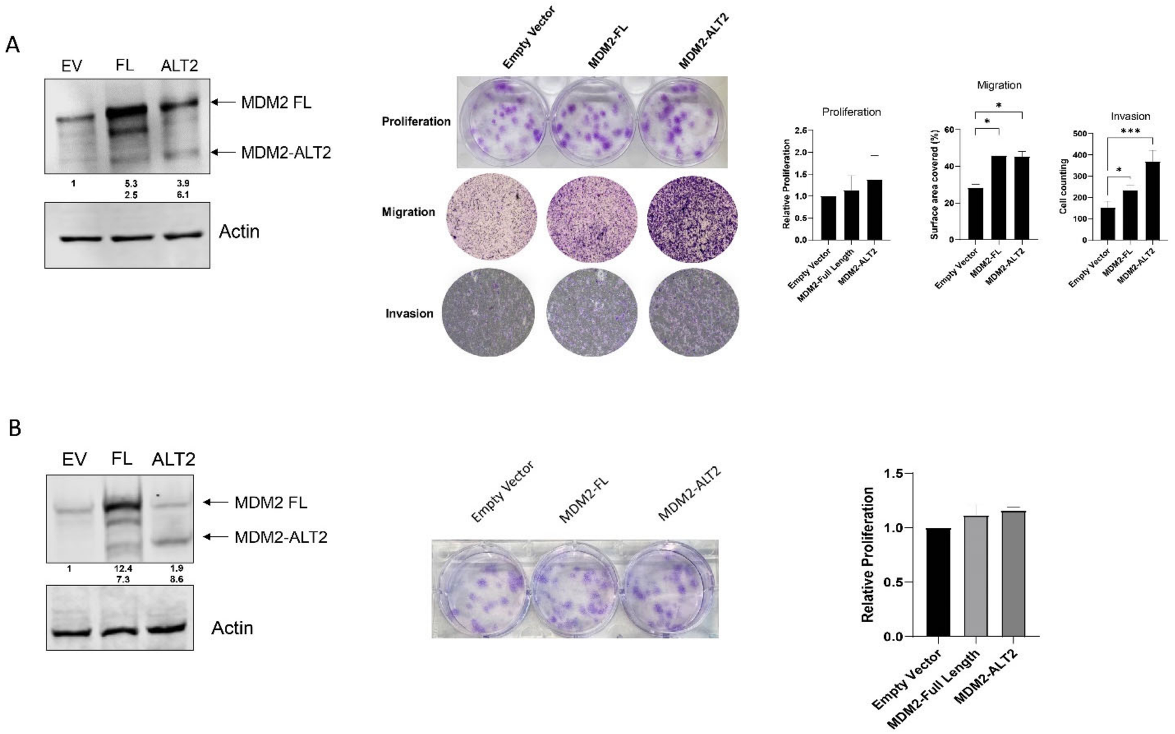

Supplementary Figure S3. LIPO246 cells overexpressing MDM2-ALT2 display an oncogenic phenotype. (**A**) MDM2-FL and MDM2-ALT2 protein levels in LIPO246 detected by Western Blot 72h after transfection and representative images and relative quantification of proliferation, migration (**p<0.05*), and invasion (**p=0.0261* and ****p=0.0007*) assays on LIPO863 cells after MDM2-FL or MDM2-ALT2 overexpression (72h). (**B**) MDM2-FL and MDM2-ALT2 protein levels and representative images of the clonogenic assay performed in SGBS cells after overexpression (72h). Images are representative of three independent experiments. EV—Empty Vector; FL—MDM2-FL; ALT2—MDM2-ALT2.

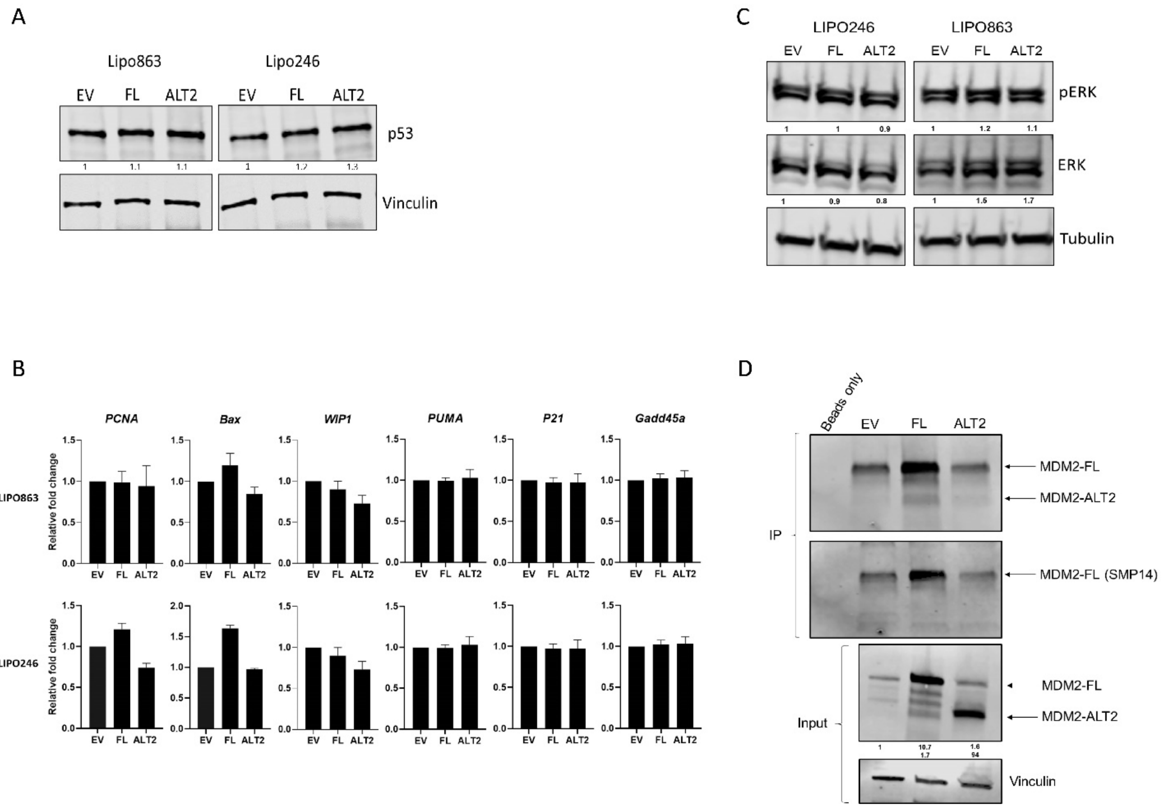

Supplementary Figure S4. MDM2-ALT2 interactions with p53 and MDM2-FL. (**A**) p53 protein levels assessed on LIPO863 and LIPO246 cells overexpressing MDM2-FL or MDM2-ALT2. The analysis was performed 72h after transfection. (**B**) mRNA relative expression levels of p53 targets determined by qPCR in RPLPS cells overexpressing MDM2-FL or MDM2-ALT2. (**C**) ERK1/2 protein levels were assessed within both LIPO863 and LIPO246 cells 72h after MDM2-FL or MDM2-ALT2 overexpression. (**D**) MDM2-FL was immunoprecipitated from transfected LIPO863 cells using an anti-MDM2 SMP14 clone antibody, then Western Blot analysis was performed to determine the presence of MDM2-FL and MDM2-ALT2 isoforms. Images are representative of three independent experiments. EV—Empty Vector; FL—MDM2-FL; ALT2—MDM2-ALT2.

A

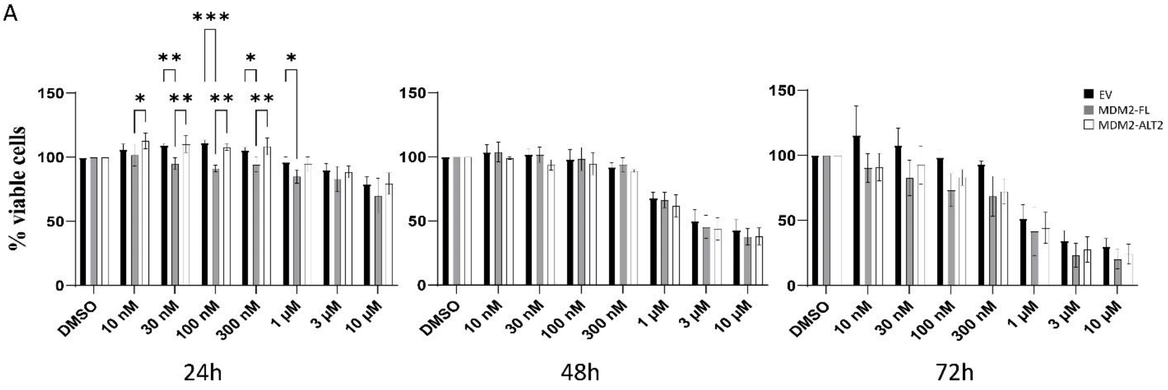

B

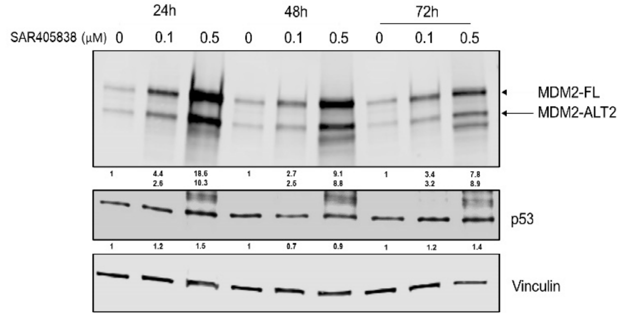

Supplementary Figure S5. Cellular sensitivity to MDM2 inhibitor treatment. (**A**) Cellular viability was assessed via MTS in LIPO863 cells overexpressing MDM2-FL or MDM2-ALT2 and treated with increasing doses of SAR405838 for 24h, 48h, and 72h. **p=0.039; **p=0.008; ***p<0.001.* (**B**) MDM2-FL and MDM2-ALT2 protein expression by Western Blot of LIPO863 after treatment with increasing doses of SAR405838. EV—Empty Vector.

A

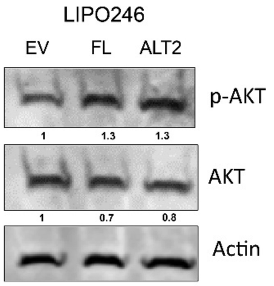

B

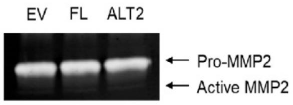

Supplementary Figure S6. (**A**) AKT and p-AKT protein levels were assessed within LIPO246 cells overexpressing MDM2-FL or MDM2-ALT2 (72h). (**B**) Gelatin zymogram was performed on LIPO863 cells to verify MMP2 activity after MDM2-FL or MDM2-ALT2 overexpression. Images are representative of three independent experiments. EV—Empty Vector; FL—MDM2-FL; ALT2—MDM2-ALT2.
